# Students' Perspectives on Curricular Ultrasound Education at German Medical Schools

**DOI:** 10.3389/fmed.2021.758255

**Published:** 2021-11-25

**Authors:** Florian Recker, Gregor Barth, Hendra Lo, Nicolas Haverkamp, Dieter Nürnberg, Dmitrij Kravchenko, Tobias Raupach, Valentin Sebastian Schäfer

**Affiliations:** ^1^Department of Obstetrics and Prenatal Medicine, University Hospital Bonn, Bonn, Germany; ^2^Brandenburg Institute for Clinical Ultrasound (BICUS), Brandenburg Medical School (Theodor Fontane), Neuruppin, Germany; ^3^Dean's Office, University Hospital Bonn, Bonn, Germany; ^4^Department of Diagnostic and Interventional Radiology, University Hospital Bonn, Bonn, Germany; ^5^Institute for Medical Education, University Hospital Bonn, Bonn, Germany; ^6^Clinic of Internal Medicine III, Department of Oncology, Hematology, Rheumatology and Clinical Immunology, University Hospital Bonn, Bonn, Germany

**Keywords:** ultrasound, ultrasound education, medical education, curriculum development, peer-teaching, medical student

## Abstract

**Background:** Despite ultrasound being an inherent part of medical education, only a few German medical schools have established a comprehensive ultrasound curriculum. This study aimed to explore medical students' perspectives on ultrasound in medical education (USMed).

**Results:** Between January 1^st^, 2019 und June 30^th^, 2019, an online survey was conducted among German medical students via the students' associations and their respective teaching facilities. The survey consisted of 17 items regarding USMed. Statements were rated on a 4-point Likert scale for agreement. In total, 1040 students from 31 German medical faculties participated. The majority (1021, 98.2%) reported a very high to high interest in curricular USMed. Students agreed (*n* = 945, 90.9%) that USMed would be helpful along their entire course of medical studies. Considering the best starting time for USMed, the opinions of German medical students diverged: students studying in a model curriculum preferred to start in the second year (40.7%) while 49% of the students studying in a traditional curriculum preferred to start in the third year (*p* ≤ 0.001). An insufficient allotment of time for USMed in the planned curriculum (675, 65%) and a lack of courses run by medical faculty (305, 29.4%) were listed as perceived significant barriers to the participation in USMed. Peer teaching was regarded as an effective method in realizing USMed by 731 (70.3%) students.

**Conclusion:** German medical students are very interested and willing to participate in USMed. There appears to be a high demand for US courses offered by medical schools.

## Introduction

For a long time, ultrasound (US) was a skill learned by physicians during their residency rather than during their studies. But in recent decades, ultrasound in medical education (USMed) has become increasingly important during medical school. In Germany, USMed as a curricular component was first introduced within the National Competency Based Catalog of Learning Objectives for Undergraduate Medical Education (NKLM) in 2015. The catalog requires students to “[be able to] use US to support basic clinical examinations according to the situation” (1) and integrates US into the curriculum in the last semesters and the practical year. However, many authors (2–5) have described that US is not just limited to diagnostic imaging, but can be used in teaching and is considered a useful additional tool for understanding complex anatomical structures and processes. In 1996, Teichgraeber et al. (6) already outlined the educational benefits of US in teaching anatomy within the preclinical curriculum. Thus, the inclusion of ultrasound in the curriculum is not only useful in terms of learning the diagnostic skill, but apparently can be supportive in other ways, such as teaching anatomy. Due to the benefits of USMed in terms of diagnostics as well as didactic support and technological advancements such as portable handheld US devices, many medical schools have started to establish USMed courses. In Germany, it is mostly offered in the form of lectures, seminars, and practical training, both on a curricular and extracurricular basis (7–9). The recently published paper from Wolf et al. (7) showed that undergraduate US courses offered at German medical schools are heterogeneous in their content and are mainly designed for advanced students. There are some medical schools that, based on many years of experience, have already realized extensive programs at both the early and advanced levels of study. There is a growing trend toward greater integration of ultrasound into medical curricula. The Universities of Erlangen-Nuremberg, Düsseldorf, and Münster offer curricular USMed for all medical students with practical training sessions over several weeks in small groups consisting of three to five medical students per tutor (10, 11). The medical faculty of the Ulm University has implemented an US course into its medical curriculum in the fifth and sixth semesters, which consists of seminars and practical training. Successful completion of a basic US course is a requirement for participation in further courses, culminating in a multiple-choice examination. Seminars are held continuously over 13 sessions covering basic subjects such as fundamentals of physics, basics of abdominal US and thyroid US, as well as more advanced subjects such as contrast-enhanced US and echocardiography. Teaching is primarily carried out by experienced specialists in internal medicine with support of student tutors (8). The Medical School Brandenburg Theodor Fontane, as one of the youngest medical schools, has integrated a curriculum for longitudinal US learning, starting in the first year of study (9). During the past years, the German Society of Ultrasound in Medicine (DEGUM) has also tried to support the integration of USMed into medical curricula. Within these efforts, a DEGUM certificate for endorsed students' education was established in 2010. It becomes apparent that there are multiple efforts to integrate USMed into medical curricula in Germany in different ways. Although there is a difference between students' perceptions of what is useful and what has proven to be beneficial to the learning process, it seems to be important to include students' perceptions in the development of medical curricula.

There is a lack of data on how beneficial the integration of ultrasound into the curriculum is perceived by students in Germany and what barriers they see regarding USMed during their studies. This study aimed to gather information on the current use and students' opinions on different points of discussion on USMed at German medical schools. Moreover, we wanted to explore the extent and type of integration USMed students consider optimal, and the barriers students perceive in learning ultrasound basic principles at their home universities.

## Methods

### Questionnaire and Distribution

An anonymous, voluntary online survey was developed to collect information regarding student opinions on USMed (see Attachment 1). Participation was possible between January 1^st^, 2019 and June 30^th^, 2019. The survey was distributed using the online platform Survio (http://www.survio.com/de/), starting with a cover letter, sent by email, including a brief description of the objectives and the purpose of the survey in cooperation with the working group of students in the German ultrasound society. The link was distributed among the local German Medical Student Councils and respective teaching facilities (skills labs) via email. Furthermore, Facebook was used to remind students via medical student groups to complete the survey. We kept track of IP addresses to prevent multiple participations. No financial reimbursement was provided.

The survey consisted of 17 questions, structured into three sections. We collected baseline characteristics, including the respective medical school, the type of medical degree program (traditional or model curriculum), and academic year in the first part of the survey. The second part consisted of six statements regarding USMed, which were to be rated on a four-point Likert scale. These included statements regarding the benefits of USMed on their medical education, and the benefits for learning and understanding physiology and spatial anatomy. In the third section, we designed multiple-choice questions with the additional option of entering a free-text answer, focusing on various aspects such as the best time to start with USMed, opinions on adequate teachers, and barriers to curricular USMed.

### Participants

At the beginning of the study in January 2019, there were 37 fully accredited German state-funded medical schools among 35 universities (12, 13). In addition, various non-governmental medical schools had been founded (for example Witten/Herdecke University, Medical School Brandenburg Theodor Fontane). To take part, participants had to study at a German medical school at the time of the survey and were requested to return a completed questionnaire.

### Data

Raw data were exported from the online platform as a Microsoft Excel Spreadsheet. Statistical analysis was performed using the software package “IBM SPSS® statistical software”, version 25.00. For each individual item we presented percentages and total number (n) of selections from all participants. The data consisted of 1040 completed questionnaires. To compare groups of students from studying in traditional and model curricula, respectively, we performed a Chi-square test to explore statistical significance. To determine the effect sizes of significant comparisons, Cramer's Phi was calculated for 2 × 2-tables and Cramer's V for all other tables.

## Results

In total, 1040 questionnaires from 31 medical schools were completed. Table 1 depicts the distribution of the number of students at each medical school. We had to exclude 28 questionnaires because of incomprehensible answers. In winter term 2018/2019, a total of 96.115 medical students were enrolled in Germany (14). Thus, 1.1% of all German medical students completed the questionnaire. Students from all academic years were represented in the questionnaire. The majority of participants were in their 3^rd^ to 5^th^ year of medical studies. Furthermore, 336 participants (32.3%) studied in a model curriculum, while 704 (67.7%) studied in a traditional curriculum.

**Table 1:** Baseline characteristics.

**Characteristics**	**Number of participants (%)**
Medical program type	
Model curriculum	336 (32.31%)
Traditional curriculum	704 (67.69%)
Academic year	
1	70 (6.92%)
2	130 (12.85%)
3	210 (20.75%)
4	233 (23.02%)
5	225 (22.23%)
6	119 (11.76%)
>7*	25 (2.48%)
Medical school of	
Philipps University Marburg	158 (15.19%)
Westfälische-Wilhelms University Münster	119 (11.44%)
Technical University Dresden	92 (8.85%)
Brandenburg Medical School Theodor Fontane	85 (8.17%)
Hannover Medical School	77 (7.4%)
Charité Medical University of Berlin	59 (5.67%)
Otto-von-Guericke University Magdeburg	48 (4.62%)
Justus-Liebig University Gießen	46 (4.42%)
Eberhard-Karls University Tübingen	37 (3.56%)
Rheinische-Friedrich-Wilhelms University Bonn	37 (3.56%)
University of Cologne	35 (3.37%)
University of Leipzig	27 (2.6%)
Carl-von-Ossietzky University Oldenburg	26 (2.5%)
University of Ulm	25 (2.4%)
University of Hamburg / UKE	25 (2.4%)
Martin-Luther University Halle-Wittenberg	25 (2.4%)
Heidelberg, Ruprecht-Karls University Heidelberg	23 (2.2%)
Mannheim, Ruprecht-Karls University Heidelberg	16 (1.5%)
Friedrich-Alexander University Erlangen-Nuremberg	15 (1.44%)
Ernst-Moritz-Arndt University Greifswald	15 (1.44%)
Albert-Ludwigs University Freiburg	11 (1.06%)
Ruhr-University Bochum	10 (0.96%)
Johannes-Gutenberg University Mainz	6 (0.58%)
University of Rostock	5 (0.48%)
Johann-Wolfgang Goethe University Frankfurt am Main	5 (0.48%)
RWTH Aachen University	4 (0.38%)
Friedrich-Schiller University Jena	3 (0.29%)
University of Regensburg	2 (0.19%)
Georg-August University Göttingen	2 (0.19%)
University of Lübeck	1 (0.1%)
University of Duisburg-Essen	1 (0.1%)

The table shows the characteristics of the participating medical students and breaks this down into the specific degree programmes, the year of study and the individual medical school. Numbers are given in total and percentage (^*^ including students who exceed the prescribed period of study or with leave of absence).

The second part of the questionnaire was designed to evaluate the participant's opinions on possible advantages of USMed and its implementation in their curricula. To explore whether students are interested in USMed, the participant's general interest in offered curricular USMed was determined (Item 5, Attachment 1). The results showed a clear tendency in favor of USMed, as 846 (81.4%) participants responded as having a very high or 175 (16.8%) high interest. To evaluate whether students would accept an increased workload, we asked for consent regarding the introduction of USMed as a compulsory course (Item 16, Attachment 1). Despite the already densely packed medical curricula, 773 participants (74.3%) stated that they strongly agree and 209 (20.1%) that they agree with the introduction of compulsory USMed into their schedules.

**Figure 1:**
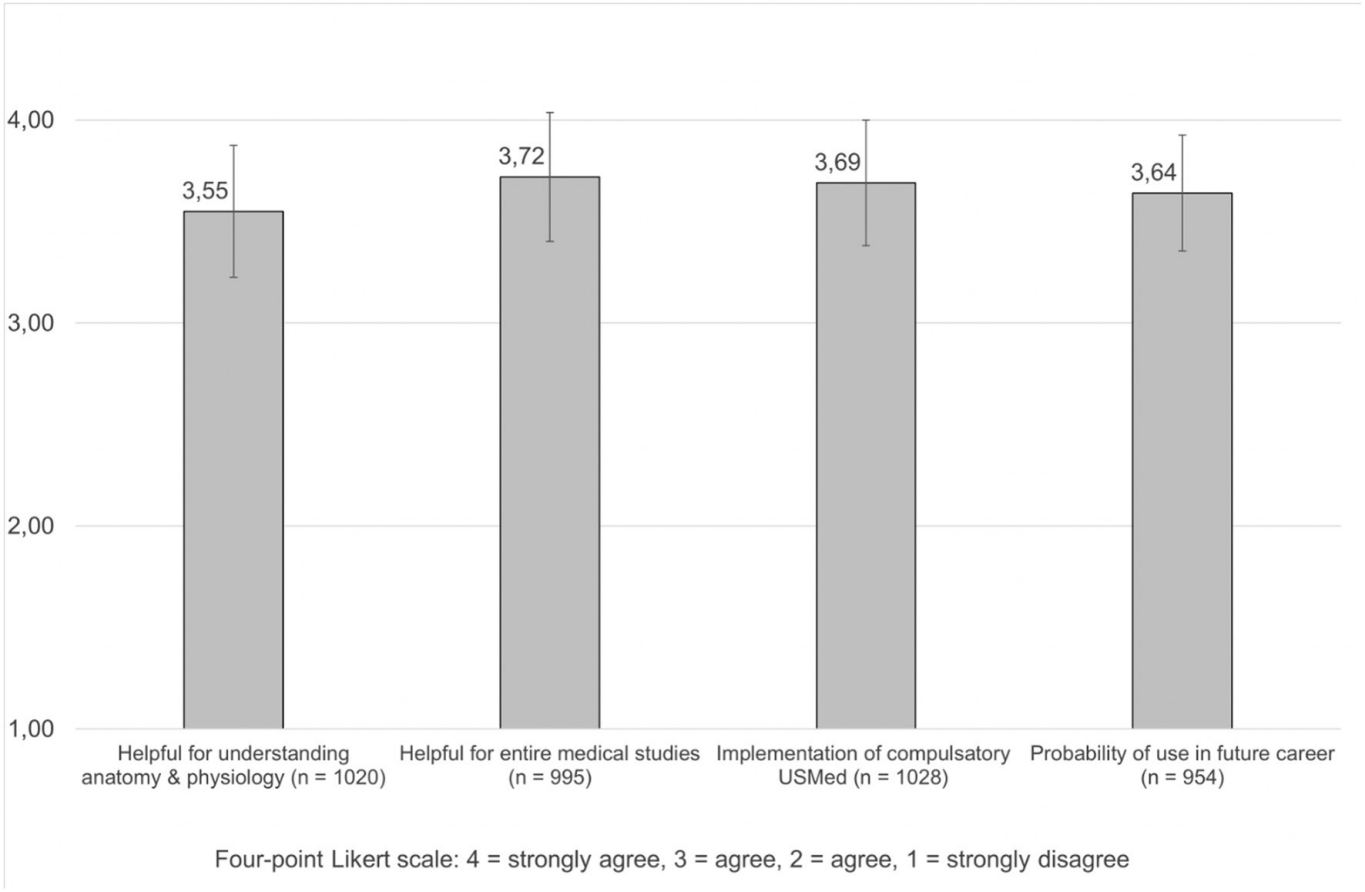
Summary of average agreement on how ultrasound in medical education (USMed) impacts medical students. Numbers are given as mean values.

Regarding the participant's opinion on whether USMed would be helpful in their medical studies, 792 (76.2%) participants ticked a one (strongly agree) on a four-point Likert scale (LS) while 153 (14.7%) responded with a two (agree). Furthermore, an overwhelming majority either strongly agreed (640 or 61.5%) or agreed (319 or 30.7%) that USMed would support their understanding of anatomy and physiology (Item 8, Attachment 1) (Figure 1).

As previously described, the last part of the survey consisted of multiple-choice questions with the possibility of free-text answers. When questioned about appropriate teachers for curricular USMed, 930 (89.4%) students believed residents and attending specialists make adequate tutors, while 731 (70.3%) found student tutors with advanced US skills just as appropriate.

**Figure 2:**
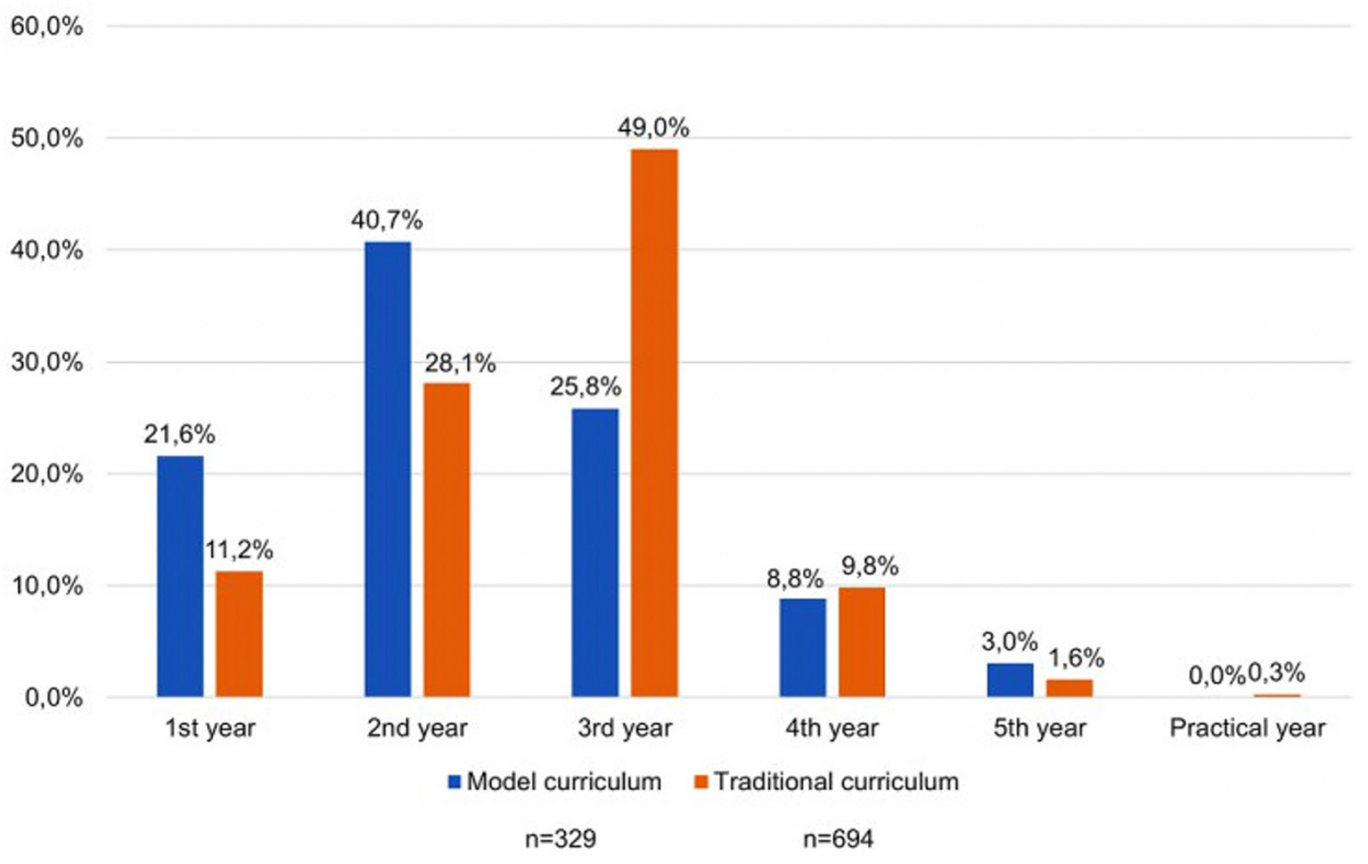
Graph depicting the best starting time for curricular ultrasound courses depending on the medical program type. Numbers are given as percentage.

The perceived ideal point in time to start USMed is displayed in Figure 2. The opinions differed between participants studying in a model curriculum and those studying in a traditional curriculum (*p* < 0.001, V = 0.242). While participants studying in a model curriculum considered the second year of study to be the most appropriate (134 or 40.7%), respondents studying in a traditional curriculum preferred the third year (340 or 49%).

**Figure 3:**
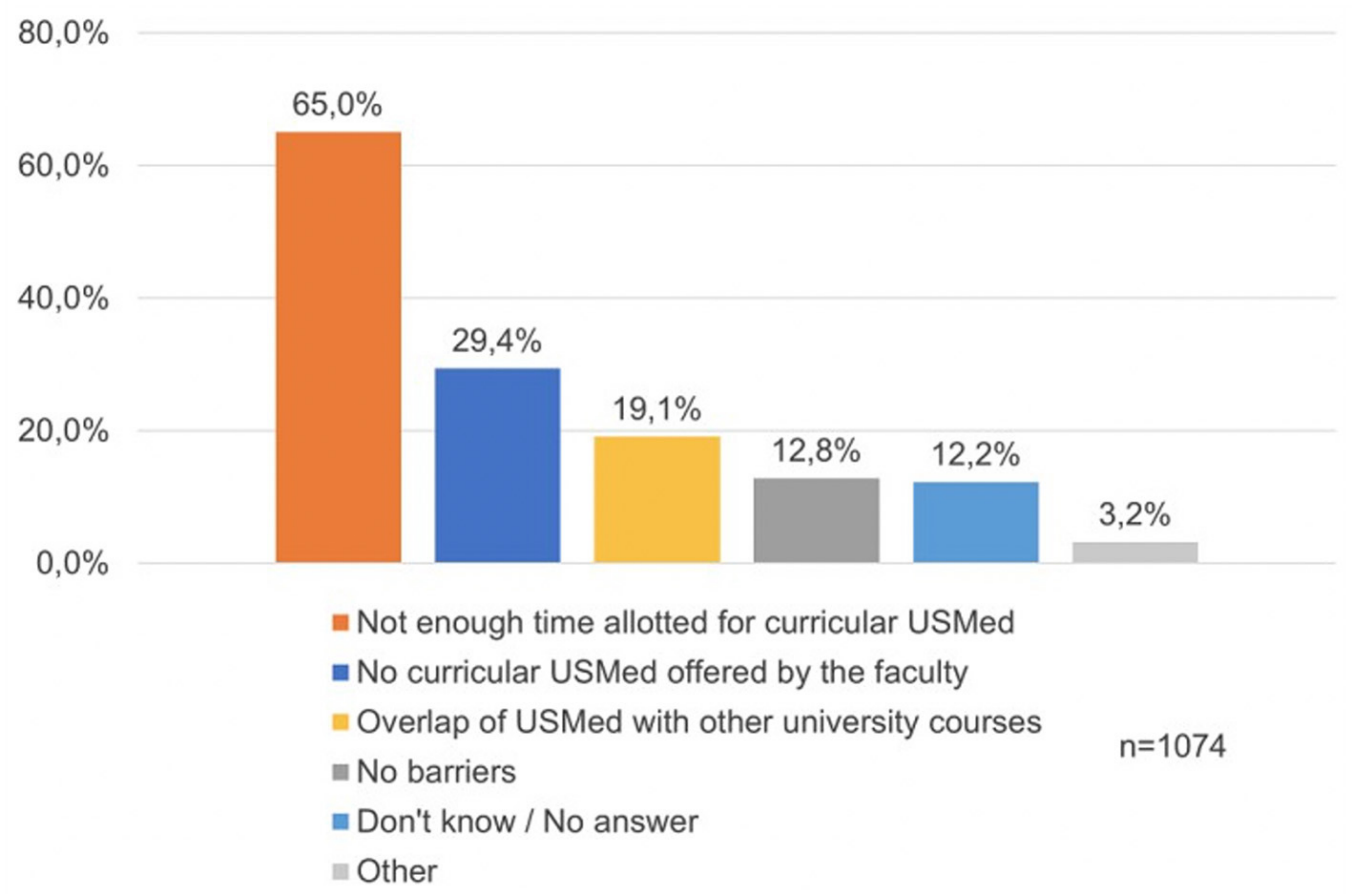
Perceived barriers for ultrasound in medical education at German medical faculties. Numbers are given as percentage.

In addition, we tried to identify perceived problems regarding the use of USMed according to individual needs or barriers that affect participation in existing USMed at medical schools (Item 14, Attachment 1). Results are summarized in Figure 3. In total, 675 (65%) participants felt there was not enough time allotted for USMed in their curricula while 305 (29.4%) noted, that their medical faculty did not offer any USMed at all. The third most commonly listed perceived barrier (*n* = 198, 19.1%) was an overlap of offered USMed with other university courses.

## Discussion

This is the first study focusing on students' perspectives on USMed in Germany. Collectively, we were able to survey over 1% of German medical students as only students from medical schools were targeted.

A recent Carnegie Foundation Report, “Educating Physicians: A Call for Reform of Medical School and Residency,” (15) stressed, that the initial years of medical education should be supported by incorporating more clinical experiences, such as anamnesis and physical examination skills. However, implementation or extension of curricular USMed results in a higher workload for students within an already highly demanding medical curriculum. Since regular use leads to an improvement of skills, it seems useful to equip students with the basic knowledge of standard US examinations as early as possible. Longitudinal curricular USMed provides a continuous transfer of knowledge and skills. As already investigated in a study by Prats et al. (16), curricular USMed seems to diminish the threshold for utilizing US examinations and shows higher rates of ultrasound use in clinical practice in residency. Further studies must assess the impact of USMed and define a reasonable extent of USMed during medical studies.

A recent systematic literature review on the value of ultrasound in undergraduate medical education revealed that there is considerable evidence that students can gain US knowledge and abilities in medical school and that they like and desire US instruction (17).

A critical question among medical students pertains to when USMed should start. Opinions differed between students studying according to the German model curriculum and the traditional degree program. Within the model curriculum, we observed a clear tendency for early integration of USMed, preferably during the second year of medical studies. This tendency could be explained by the more practical nature of this study program. Participants at the Medical School Brandenburg Theodor Fontane were the only students studying in a model curriculum who clearly preferred the first year of medical school as an ideal starting point for USMed, as this rather young university offers a practice-oriented curriculum with an early integration of clinical and imaging skills.

In contrast to the participants' opinions, many other authors have already shown, that implementation of USMed seems to be useful in a preclinical context to support learning processes and visualization of complex contents (4, 10). Hofer et al. (11) implemented USMed in their medical curriculum at the university of Düsseldorf in 1996. They reported on the usefulness of implementing USMed in preclinical semesters and described, that reasonable integration of USMed in the curriculum can lead to optimization of teaching and skills.

The WFUMB has already published its position paper on this topic and described critical components of the integration of US into medical curricula (18). Germany however, still lacks concrete national learning objectives that can provide medical schools with guidance on what is expected from students in terms of US skills. The current learning objectives are only vague and do not meet the requirements for precise training in sonography with a focus on the abdomen in the current version of the national competence curriculum.

Numerous participants indicated concerns regarding the sparsely offered US courses in their curricula. In addition, the majority described a lack of allotted time for additional US courses due to collisions with other curricular activities (Figure 3). Participants often reported limited availability of course placements due to high demand. Although there appears to be a consensus among students that there is a wish for integration of US into the medical curriculum, there are clear delineated barriers that currently limit further adoption. However, in order to create a learner-friendly atmosphere and to enable students to get the most benefits out of an US course, it is necessary to design US courses in a way that students can use them according to their needs. As shown in other studies (11, 19), it is possible to design high-quality USMed despite high demand and additional costs. Wolf et al. (7) suggested that one possibility would be to use peer tutors. The majority of participants (731 or 70.3%) stated that student US tutors with advanced US skills are suitable alternatives to residents or specialists. In regard, the use of peer tutors in ultrasound education is demonstrated in numerous studies (7, 20–24). Peer-teaching seems to be a widely accepted method by medical students with the advantage of knowledge transfer at the same level and highly motivated teachers, enabling exercises in small groups rather than fully packed courses (25, 26). Student tutors can of course not replace the lecturing and supervision of an experienced physician, but rather complement them (27). At present, the training of US tutors at German medical schools is heterogeneous with several different existing approaches for US tutor training (28). But in order to establish minimum USMed standards to guarantee a certain quality of teaching throughout Germany, specific learning objectives should be integrated and added to the upcoming NKLM in this effort to provide in-depth ultrasound education nationally. These will enable medical schools to develop their specific USMed programs and to demonstrate and evaluate the effectiveness of these programs with validated test methods based on the achievement of standardized learning objectives.

USMed has been proven to be a feasible and well-accepted goal by medical students. In a recently published study, authors showed, that after only five hours of instruction, it has the ability to assist medical students gain basic competency in abdominal ultrasound examination (29). Many studies (30–32) have looked at the use of the Objective Structured Clinical Examination (OSCE), with Todsen et al. (31) demonstrating strong reliability and evidence of construct validity of the OSAUS scale in an educational setting. The question on whether to start a longitudinal ultrasound curriculum in a preclinical or clinical section of the medical curriculum remains to be answered.

One of the limitations of this study is the possibility of a non-response bias and a self-selection bias due to voluntary participation. Only about one percent of all medical students participated in the survey which limits our ability to draw conclusions. Since the survey was accessible to everyone via an internet link, we cannot guarantee that only medical students participated in the survey. Another limitation is the positive response in such studies from students who strongly favor current and practical topics. However, it is often overlooked that there must also be examinations for all compulsory courses and that in most cases other parts must be deleted from the curriculum. Furthermore, it is possible that participants completed more than one survey from different computers. We tried to reduce this problem by limiting the number of participations per IP address.

In summary, medical students are in favor of an integration and intensification of USMed offerings, which are considered useful throughout their studies. The ideal point in time to introduce USMed should be determined from a didactical point of view, taking into account the specifics of the curriculum of each faculty. There is a need for the development of national standards in order to facilitate widespread adoption of US education in German medical curricula. Current barriers to the use of USMed are mainly seen in the insufficient curricular time allotted for US courses and the general lack of courses and course placements.

## Data Availability Statement

The raw data supporting the conclusions of this article will be made available by the authors, without undue reservation.

## Ethics Statement

The studies involving human participants were reviewed and approved by Ethics Committee of the Medical Faculty of the Rheinische Friedrich-Wilhelms-Universität Bonn.

## Author Contributions

All authors contributed to the study conception and design. Material preparation, data collection and analysis were performed by GB, FR, and NH. The first draft of the manuscript was written by GB, FR, and VS and all authors commented on previous versions of the manuscript. HL, DN, DK, and TR helped by manuscript editing. All authors read and approved the final manuscript.

## Conflict of Interest

The authors declare that the research was conducted in the absence of any commercial or financial relationships that could be construed as a potential conflict of interest.

## Publisher's Note

All claims expressed in this article are solely those of the authors and do not necessarily represent those of their affiliated organizations, or those of the publisher, the editors and the reviewers. Any product that may be evaluated in this article, or claim that may be made by its manufacturer, is not guaranteed or endorsed by the publisher.
