# Supplementary material for: Students' Perspectives on Curricular Ultrasound Education at German Medical Schools
Source: Front Med (Lausanne). 2021 Nov 25;8:758255. doi: 10.3389/fmed.2021.758255 (PMC8655332; doi:10.3389/fmed.2021.758255)
Supplement: Supplementary file 1 [file Data_Sheet_1.PDF]

Universitätsklinikum Bonn
Venusberg-Campus 1
53127 Bonn

Demand for ultrasound training among medical students in Germany

1. City:

2. College:

3. Year of study:

4. Study organization: regular study ☐ Model study program ☐

5. My interest in a university offered curricular ultrasound class is...

Very high ☐ Rather high ☐ Rather low ☐ I have no interest in such an offer ☐

6. I would also take advantage of the offer of university offered curricular ultrasound instruction if participation is voluntary.

I totally agree ☐ I rather agree ☐ I rather disagree ☐ I disagree ☐ I cannot judge ☐

7. To gain practical skills of sonography during my study, I would... (Multiple selection possible)

Attend curricular courses offered by the university with mandatory attendance. ☐

Attend curricular courses offered by the university with voluntary participation. ☐

Attend non-university courses (conferences, sono schools, private courses, conventions). ☐

Other:

8. I think that ultrasound classes offered by my university would enhance my understanding in the subjects of anatomy and physiology.

I totally agree ☐ I rather agree ☐ I rather disagree ☐ I disagree ☐ I cannot judge ☐

Universitätsklinikum Bonn
Venusberg-Campus 1
53127 Bonn

UNIVERSITÄT BONN ukb universitäts klinikumbonn

9. from my university curricular - offered ultrasound classes are in my opinion helpful for my medical studies.

I totally agree ☐ I rather agree ☐ I rather disagree ☐ I disagree ☐ I cannot judge ☐

10. To learn theoretical knowledge of sonography during my studies, I would... (multiple selection possible)

- Attend curricular courses offered by the university with mandatory attendance. ☐
- Attend curricular courses offered by the university with voluntary participation. ☐
- Attend extracurricular courses offered by the university. ☐
- Attend non-university courses (conferences, sono schools, private courses, conventions). ☐

Other:

11. I think in curricular offered ultrasound lessons would be suitable as lecturers/trainers: (multiple choice possible).

- Students with advanced skills (ultrasound tutors) ☐
- Specialists and doctors in training ☐
- I cannot judge ☐

12. To participate for the first time in an ultrasound course offered by my university would be useful for me:

- The 1st and/or 2nd semester ☐
- The 3rd and/or 4th semester ☐
- The 5th and/or 6th semester ☐
- The 7th and/or 8th semester ☐
- The 9th and/or 10th semester ☐
- In the practical year ☐
- I cannot judge ☐

Universitätsklinikum Bonn
Venusberg-Campus 1
53127 Bonn

13. On whom do you train your practical skills in ultrasound during the curricular university courses?
(Multiple selection possible)

Patients ☐ Simulators ☐ Students ☐ Models / Participants ☐

Other:

14. Which hurdles do you train your practical skills in ultrasound during curricular university courses?
(Multiple selection possible)

- Too little curricular planned time for ultrasound training ☐
- Overlap with other important courses of the university ☐
- I see no hurdles ☐
- I cannot judge ☐

Other:

15. Where do you acquire theoretical knowledge on the subject of ultrasound?
(Multiple selection possible)

- Lectures at your own university ☐
- Seminars at your own university ☐
- Lectures at another university ☐
- Seminars at another university ☐
- Lectures/courses at congresses, and from private providers or similar. ☐
- Books/Magazines ☐
- E-learning (Internet) ☐
- Apps ☐
- Not at all ☐

Other:

16. I think ultrasound teaching should be offered by the university as a compulsory course.

I totally agree ☐ I rather agree ☐ I rather disagree ☐ I disagree ☐ I cannot judge ☐

17. through the curricular ultrasound teaching offered by the university (own or others), I think it is likely that I will use ultrasound later in my professional life.

I totally agree ☐ I rather agree ☐ I rather disagree ☐ I disagree ☐ I cannot judge ☐
