# Supplementary material for: Students' Perspectives on Curricular Ultrasound Education at German Medical Schools
Source: Front Med (Lausanne). 2021 Nov 25;8:758255. doi: 10.3389/fmed.2021.758255 (PMC8655332; doi:10.3389/fmed.2021.758255)
Supplement: Supplementary file 2 [file Data_Sheet_2.PDF]

Nachfrage Medizinstudierender in Deutschland nach Ultraschall-Ausbildung

1. Stadt:

2. Hochschule:

3. Studienjahr:

4. Studiumsorganisation: □ Regelstudiengang □ Modellstudiengang

5. Mein Interesse an einem universitär angebotenen curricularen Ultraschallunterricht ist…

□	Sehr hoch
□	Eher hoch
□	Eher gering
□	Ich habe kein Interesse an so einem Angebot

6. Ich würde das Angebot universitär angebotenen curricularen Ultraschallunterrichts auch nutzen, wenn die Teilnahme freiwillig ist.

□	Stimme voll zu
□	Stimme eher zu
□	Stimme eher nicht zu
□	Stimme nicht zu
□	Kann ich nicht beurteilen

7. Um während meines Studiums praktische Fähigkeiten der Sonographie zu erlangen, würde ich … (Mehrfachauswahl möglich)

□	von der Uni angebotene curriculare Kurse mit Pflichtteilnahme besuchen.
□	von der Uni angebotene curriculare Kurse mit freiwilliger Teilnahme besuchen.
□	außeruniversitäre Kurse (Konferenzen, Sonoschulen, Privatkurse, Kongresse) besuchen.
□	Sonstiges:

8. Ich denke, dass von meiner Hochschule curricular - angebotener Ultraschallunterricht mein Verständnis in den Fächern Anatomie und Physiologie fördern würde.

□	Stimme voll zu
□	Stimme eher zu
□	Stimme eher nicht zu
□	Stimme nicht zu
□	Kann ich nicht beurteilen

9. Von meiner Hochschule curricular - angebotener Ultraschallunterricht ist meines Erachtens nach für mein Medizinstudium hilfreich.

□	Stimme voll zu
□	Stimme eher zu
□	Stimme eher nicht zu
□	Stimme nicht zu
□	Kann ich nicht beurteilen

10. Um während meines Studiums theoretische Kenntnisse der Sonographie zu erlernen, würde ich … (Mehrfachauswahl möglich)

□	von der Uni angebotene curriculare Kurse mit Pflichtteilnahme besuchen.
□	von der Uni angebotene curriculare Kurse mit freiwilliger Teilnahme besuchen.
□	von der Uni angebotene außercurriculare Kurse besuchen.
□	außeruniversitäre Kurse (Konferenzen, Sonoschulen, Privatkurse, Kongresse) besuchen.
□	Sonstiges:

11. Ich denke, in curricular angebotenem Ultraschallunterricht würden sich als Dozenten/Trainer eignen: (Mehrfachauswahl möglich)

□	Studierende mit erweiterten Fähigkeiten (Ultraschalltutoren)
□	Fachärzte und Ärzte in Ausbildung
□	Kann ich nicht beurteilen

12. Erstmalig an einem von meiner Hochschule curricular - angebotenen Ultraschallunterricht teilzunehmen wäre für mich sinnvoll ab:

□	Dem 1. und/oder 2. Fachsemester
□	Dem 3. und/oder 4. Fachsemester
□	Dem 5. und/oder 6. Fachsemester
□	Dem 7. und/oder 8. Fachsemester
□	Dem 9. und/oder 10. Fachsemester
□	Im Praktischen Jahr
□	Kann ich nicht beurteilen

13. An wem trainieren Sie während der curricular universitären Kurse Ihre praktischen Fertigkeiten im Ultraschall? (Mehrfachauswahl möglich)

□	Patienten
□	Simulatoren
□	Studierenden
□	Models / Teilnehmer
Andere:	

14. Welche Hürden sehen Sie derzeit, die es Ihnen erschweren, das universitäre Angebot des Ultraschallunterrichtes der Ihren Bedürfnissen zu nutzen? (Mehrfachauswahl möglich)

□	Zu wenig curricular geplante Zeit für die Ultraschallausbildung
□	Keine Angebote der Universität zur Ultraschallausbildung
□	Überschneidung mit anderen wichtigen Lehrveranstaltungen der Hochschule
□	Ich sehe keine Hürden
□	Kann ich nicht beurteilen
Andere:	

15. Wo erwerben Sie theoretischen Wissen zum Thema Ultraschall? (Mehrfachauswahl möglich)

◻	Vorträge in der eigenen Hochschule
◻	Seminare in der eigenen Hochschule
◻	Vorträge an einer anderen Hochschule
◻	Seminare an einer anderen Hochschule
◻	Vorträge/Kurse auf Kongressen, von privaten Anbietern o.ä.
◻	Bücher/Zeitschriften
◻	E-Learning (Internet)
◻	Apps
◻	Gar nicht
Andere:	

16. Ich finde, Ultraschallunterricht sollte von der Universität als Pflichtveranstaltung angeboten werden.

◻	Stimme zu
◻	Stimme eher zu
◻	Stimme eher nicht zu
◻	Stimme nicht zu
◻	Kann ich nicht beurteilen

17. Durch den von der Hochschule (eigene oder fremde) angebotenen curricularen Ultraschallunterricht, halte ich es für wahrscheinlich, dass ich Ultraschall später in meinem Berufsalltag nutzen werde.

◻	Stimme zu
◻	Stimme eher zu
◻	Stimme eher nicht zu
◻	Stimme nicht zu
◻	Kann ich nicht beurteilen
